# Sociodemographic risk factors for the persistence of harmful alcohol use: a pooled analysis of prospective cohort studies

**DOI:** 10.1007/s00127-024-02654-w

**Published:** 2024-04-26

**Authors:** Anna Ehrnrooth, Kia Gluschkoff, Markus Jokela, Kaisla Komulainen

**Affiliations:** Department of Psychology and Logopedics, University of Helsinki, P.O. Box 21, 00014 Helsinki, Finland

**Keywords:** Persistent harmful alcohol use, Sociodemographic risk factors, Meta-analysis, Prospective cohort studies

## Abstract

**Purpose:**

Previous research suggests several sociodemographic risk factors for the persistence of harmful alcohol use. However, the evidence is limited due to short follow-up times, retrospective reporting and samples comprising only people with alcohol dependence. We pooled data from six prospective cohort studies to systematically evaluate whether the sociodemographic risk factors differ between the incidence and persistence of harmful alcohol use.

**Methods:**

Data were from six prospective cohort studies from the US, UK and Japan (n = 28,394). We conducted a two-stage meta-analysis to examine the associations of six sociodemographic risk factors (sex, age, presence of a partner, educational attainment, smoking and psychological distress) with the incidence and persistence of harmful alcohol use. Tests of heterogeneity were used to evaluate whether the associations differ between the incident and persistent use.

**Results:**

Male sex, younger age, higher education, smoking, and psychological distress were associated with a greater risk of both the incidence and the persistence of harmful alcohol use in mutually adjusted models (ORs = 0.97–1.67). There were no differences in the associations of these risk factors with incident and persistent use, except that the association of psychological distress was greater with incident use compared to persistent use (p for heterogeneity < 0.05).

**Conclusions:**

These findings suggest that the incidence and persistence of harmful alcohol use share a similar set of sociodemographic risk factors in the general population.

**Supplementary Information:**

The online version contains supplementary material available at 10.1007/s00127-024-02654-w.

## Introduction

Alcohol dependence (as defined by the DSM-5 criteria)—and persistent harmful alcohol use even at subclinical levels—is a chronic mental health problem that increases the risk of serious health conditions [19].Although 75% to 91% of alcohol dependent individuals eventually recover, relapses are common and full recovery is slow [1, 3, 13, 14, 21]. To improve recovery, it is important to identify risk factors for the persistence of harmful alcohol use.

Previous research suggests that the persistence of harmful alcohol use is associated with male sex, younger age, absence of a partner, ethnic minority status, higher educational attainment, smoking and psychological distress [1–3, 5, 11, 13–15]. However, some of the findings have been mixed. For instance, younger age has also been associated with lower likelihood of persistent harmful alcohol use, and some studies have reported no association, or even negative association, with higher educational attainment and incidence or persistence of harmful alcohol use [3, 11],Moos & Moos, n.d.). Moreover, many of the studies have been limited by retrospective study designs, and the few longitudinal studies are limited to short (2–3 years) follow-ups and small samples comprising of people who initially met the criteria for alcohol dependence, rather than representing the general population [1, 3, 4, 23].

In addition to the persistence of harmful alcohol use, several studies have shown that male sex, younger age, absence of a partner, ethnic minority status, lower educational attainment, smoking and psychological distress are associated with the incidence of harmful alcohol use [7, 9, 11],Lopez-Quintero, Pérez De Los Cobos, et al., 2011; [22].

This suggests that similar risk factors may contribute to both incidence and persistence of harmful alcohol use. However, whether some sociodemographic factors are more important for persistence than incidence has not been systematically compared. We pooled data from six prospective cohort studies and examined how sociodemographic risk factors (sex, age, presence of a partner, educational attainment, smoking status, psychological distress) are associated with the incidence and persistence of harmful alcohol use in the general population. The longitudinal data allowed us to examine whether these risk factors were differently associated with the persistence versus incidence of harmful alcohol use.

## Material and methods

### Subjects

We identified eligible large-scale cohort studies on alcohol use and sociodemographic factors by searching the collections of the UK Data Service (https://ukdataservice.ac.uk) and the Inter-University Consortium for Political and Social Research (http://www.icpsr.umich.edu/icpsrweb/ICPSR/). Eligible studies were selected based on three criteria: first, studies provided individual-level data for adults; second, studies used validated assessment methods to measure alcohol use and sociodemographic risk factors; and third, studies had a prospective design with at least two waves of data collection.

**Fig. 1:**
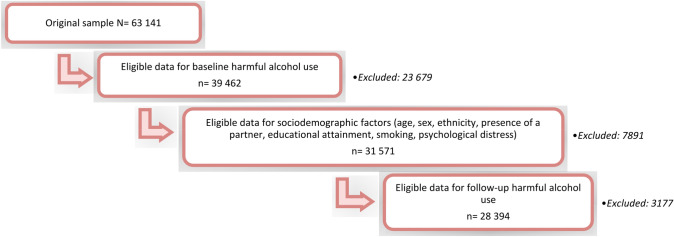
Selection of the final study sample

We identified six population-based cohort studies: the Wisconsin Longitudinal Study Graduate and Sibling samples (WLSG and WLSS), the Midlife in the United States Study (MIDUS), the Midlife in Japan Study (MIDJA), the National Child Development Study (NCDS), and the British Birth Cohort Study (BCS) (See Table 1 for the descriptive characteristics of the study populations). The original samples of the studies included 63 141 participants in total. Of the 31 571 participants who provided data on alcohol use and sociodemographic factors at baseline, 3177 were excluded due to missing data on alcohol use at follow-up, yielding a final study sample of 28 394 (Fig. 1). All participants provided informed consent for participation.

**Table 1:** Descriptive characteristics of the study population (N = 28394)

Sociodemographic factor	Harmful alcohol use at baseline (n = 5575)	No harmful alcohol use at baseline (n = 22819)
Sex (male)	3453 (62%)	9922 (43%)
Age*	38.8 (10.5)	38.6 (11.0)
Presence of a partner (not partnered)	1599 (29%)	5073 (22%)
Educational attainment		
Primary	835 (15%)	3691 (16%)
Secondary	2821 (51%)	12,084 (53%)
Tertiary	1919 (34%)	7044 (31%)
Smoking status (current smoker)	2025 (36%)	5820 (26%)
Psychological distress (z-score)*	0.1 (1.1)	-0.1 (0.9)
Harmful alcohol use at follow-up	3263 (59%)	2992 (13%)

Values are frequencies (%) unless otherwise indicated

*Mean (SD)

### Assessment of harmful alcohol use

Harmful alcohol use was evaluated at each study baseline and at follow-up with self-report questionnaires. Harmful alcohol use was assessed with the *CAGE questionnaire* (BCS and NCDS), or a 5-item composite (MIDUS and WLS) or a 2-item composite (MIDJA) similar to CAGE (see Supplementary material for the items in each cohort). The acronym CAGE stands for Cut, Annoyed, Guilty, and Eye that represent the four questions of the screening scale: Have you ever felt you ought to *cut down* on drinking? Have people *annoyed* you by criticising your drinking? Have you ever felt bad or *guilty* about your drinking? Have you ever had a drink first thing in the morning (*eye*-*opener*) to steady your hands? The responses to the questionnaire items were dichotomized into harmful alcohol use (if the participant answered “yes” to at least 1 item) vs. no harmful alcohol use (if the participant answered “no” to all items). Persistent harmful alcohol use was inferred from meeting at least one of the CAGE risk criteria both at baseline and follow-up.

### Assessment of sociodemographic factors

Six sociodemographic factors were assessed at each study baseline through participants’ self-reports. These were age (in years), sex (0 = female, 1 = male), presence of a partner (0 = partnered, 1 = not partnered), educational attainment (1 = primary, 2 = secondary, 3 = tertiary), current smoking status (0 = never smoked or ex-smoker, 1 = current smoker) and psychological distress. Descriptions of coding of the variables in each dataset is presented in Supplementary Table 2. Psychological distress was assessed with the General Health Questionnaire, (BCS), Center for Epidemiologic Studies Depression Scale (WLSG, WLSS), the Malaise inventory (NCDS), Negative Affectivity Scale (MIDJA) and K-6 Distress Scale (MIDUS). Psychological distress scores were standardised (z-scores) for the analyses. In MIDUS, we also assessed ethnicity (0 = ethnic majority, 1 = ethnic minority).

### Statistical analyses

We followed a two-stage meta-analytical approach to evaluate the associations of sociodemographic factors with harmful alcohol use. The associations of all sociodemographic factors with harmful alcohol use at follow-up were examined simultaneously in mutually adjusted models, and the reported results are therefore the associations of each sociodemographic factor with harmful alcohol use when controlling for all other sociodemographic factors. All analyses were also adjusted for the length of follow-up. Compared to the other included cohorts, MIDUS comprised participants from more ethnically diverse backgrounds, and we additionally adjusted for ethnic minority status in analyses conducted in MIDUS. To assess the *incidence* of harmful alcohol use, we conducted a series of logistic regression analyses among those with no harmful alcohol use at baseline, where we predicted harmful alcohol use at follow-up with each sociodemographic factor at baseline (age, sex, presence of a partner, educational attainment, current smoking status, and psychological distress). These analyses were conducted separately in each six studies, and the estimates obtained from these analyses were pooled in a random-effects meta-analysis. To assess the *persistence* of harmful alcohol use, we conducted a series of logistic regression analyses among those with harmful alcohol use at baseline, where we predicted harmful alcohol use at follow-up with the sociodemographic factors at baseline. As in the analysis for incidence, the analyses for persistence were conducted separately in each six studies, and the estimates obtained from these analyses were pooled in a random-effects meta-analysis. To compare the pooled estimates between those with no harmful alcohol use at baseline (the incidence of harmful alcohol use) and those with harmful alcohol use (the persistence of harmful alcohol use) at baseline, we conducted tests of heterogeneity. To illustrate the associations, we plotted the marginal predicted probabilities for the persistence of harmful alcohol use at different levels of the sociodemographic risk factors.

In each cohort, we conducted attrition analyses where attrition at follow-up was predicted by baseline harmful alcohol use status.The analyses were performed using STATA 17 statistical software.

## Results

Characteristics of the pooled sample are presented in Table 1. The mean age in the pooled sample was 39 (SD = 11), and there were 15 019 (53%) women. In total, 5 575 (20%) participants reported harmful alcohol use at baseline and 6 255 (22%) reported harmful alcohol use at follow-up. Among those with no harmful alcohol use at baseline, 13% reported incidence of harmful alcohol use at follow-up; and of those with harmful alcohol use at baseline, 59% reported persistence of harmful alcohol use at follow-up. Follow-up times ranged from 4 to 19 years between the included studies, with a mean follow-up time of 8 years.

Harmful alcohol use at baseline predicted attrition in one of the cohorts, BCS (p < 0.001, OR 0.27, 95% CI 0.13, 0.58, see Supplementary Table 2).

**Fig. 2:**
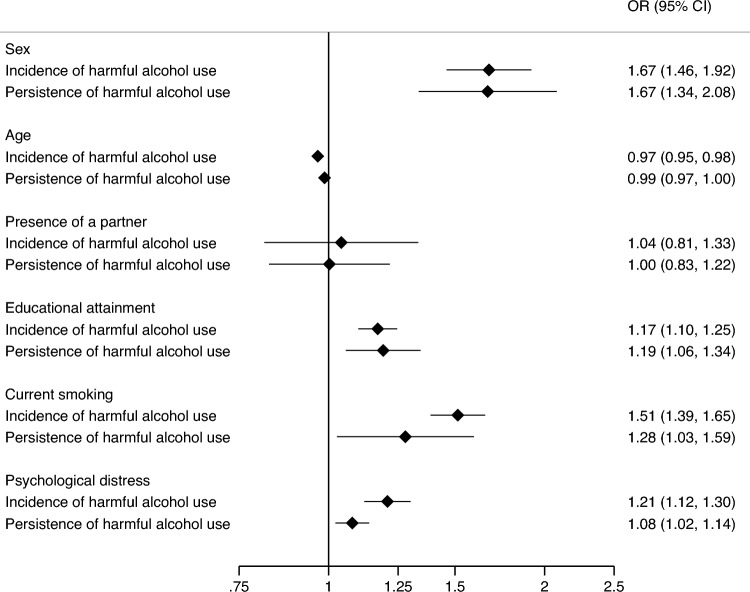
Associations of sociodemographic risk factors with the incidence and persistence of harmful alcohol use in all six data sets combined*.* Estimates for the incidence of harmful alcohol use are from analyses among participants with no harmful alcohol use at baseline. Estimates for the persistence of harmful alcohol use are from analyses among participants with harmful alcohol use at baseline. All analyses adjusted for the length of follow-up and the other sociodemographic factors of interest (analyses in MIDUS additionally adjusted for ethnic minority status). Presence of a partner was coded 0 = partnered, 1 = not partnered)

The associations of sociodemographic risk factors with harmful alcohol use are presented in Fig. 2. Among those with no harmful alcohol use at baseline, male sex (OR 1.67, 95% CI 1.46, 1.92), younger age (OR 0.97, 95% CI 0.95, 0.98), higher educational attainment (OR 1.17, 95% CI 1.10, 1.25), current smoking (OR 1.51, CI 1.39, 1.65) and psychological distress (OR 1.21, CI 1.12, 1.30) were associated with the incidence of harmful alcohol use at follow-up (see Supplementary Figs. 1–6). Similarly, among those with harmful alcohol use at baseline, male sex (OR 1.67, CI 1.34, 2.08), younger age (OR 0.99, CI 0.97, 1.00), higher educational attainment (OR 1.19, CI 1.06, 1.34), current smoking (OR 1.28, CI 1.03, 1.59) and psychological distress (OR 1.08, CI 1.02, 1.14) were associated with the persistence of harmful alcohol use at follow-up (see Supplementary Figs. 1–6).Presence of a partner was not associated with harmful alcohol use at follow-up either among those with no harmful alcohol use at baseline or those with harmful alcohol use at baseline.

**Fig. 3:**
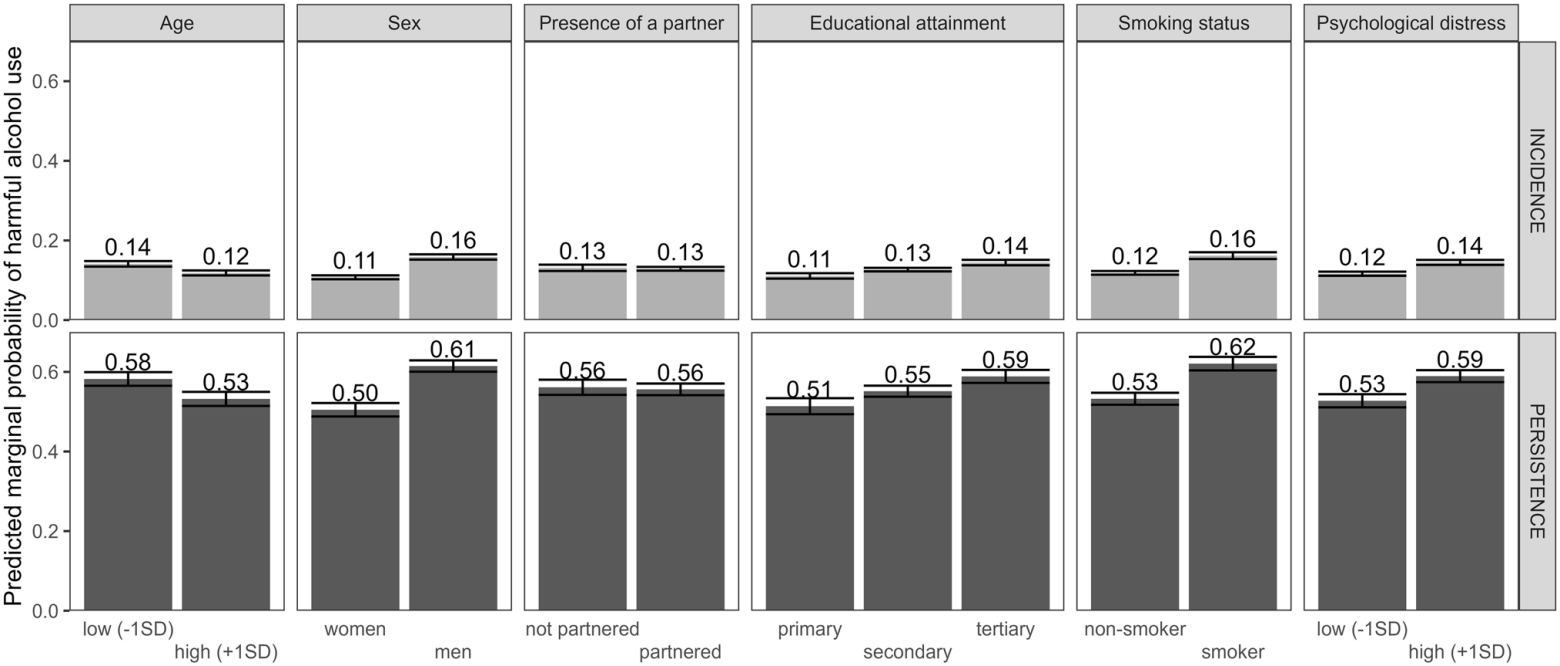
Marginal probabilities for the incidence and persistence of harmful alcohol use at different levels of sociodemographic risk factors

The heterogeneity tests indicated that the associations of sociodemographic risk factors were similar for both incident and persistent harmful alcohol use (p-values for heterogeneity > 0.05, Fig. 2, Supplementary Figs. 1–5), except that the association of psychological distress with incident harmful alcohol use was greater compared to persistent harmful alcohol use (p = 0.016, Fig. 2, Supplementary Fig. 6). Figure 3 presents the marginal probabilities of the incidence and persistence of harmful alcohol use at different levels of sociodemographic risk factors.

## Discussion

Pooling data from six prospective cohort studies, we observed that both the incidence and persistence of harmful alcohol use were associated with a similar set of sociodemographic risk factors: male sex, younger age, higher educational attainment, current smoking and psychological distress were associated with greater likelihood of both the incidence and persistence of harmful alcohol use in the general population. There were no differences in the risk factors between the incidence and persistence of harmful alcohol use, except for the association of psychological distress which was greater with incident harmful alcohol use compared to persistent harmful alcohol use.

Our findings are generally in line with previous studies suggesting that male sex, younger age, smoking, and psychological distress increase the risk for both incidence and persistence of harmful alcohol use. [1–3, 5],Gärant et al., [11, 13, 14],Lopez-Quintero, Pérez De Los Cobos, et al., [15, 22]. In our study, male sex and current smoking had the strongest associations with both the incidence and persistence of harmful alcohol use. The association of age with incident harmful alcohol use was small, and the association with persistent use was close to null. Previous findings regarding the association of age with persistent alcohol use have been mixed [11], with younger age associated with both higher and lower risk of persistent harmful alcohol use. It is possible that the association of age and harmful alcohol use is nonlinear, which could be reflected in our findings. Future studies are needed to evaluate the potential nonlinear associations between age and harmful alcohol use.

Our findings suggest that higher educational attainment is associated with both incident and persistent harmful alcohol use. In contrast to our results, some previous studies have linked higher educational attainment with lower likelihood of the incidence and persistence of alcohol dependence [11, 22]. A recent study suggested that higher educational attainment is associated with higher frequency of alcohol use, but lower risk for alcohol dependence [20]. The’alcohol harm paradox’ refers to the discrepancy in the harm caused by alcohol consumption between lower and higher socio-economic groups [10, 12]: although highly educated people may consume alcohol at harmful levels, they may be less likely to meet the diagnostic criteria for alcohol dependence. Our findings are in line with this hypothesis since we defined harmful alcohol use as having at least one of the alcohol use questionnaire items answered “yes”, which yields a low-threshold measure for harmful alcohol use, and the persistence of harmful alcohol use among highly educated people may be related to the lower harm and thus fewer clinical interventions or less incentive to stop drinking. Contrary to previous studies, the presence of a partner was not associated with the incidence nor the persistence of harmful alcohol use. Given that previous findings regarding the presence of a partner have been relatively consistent, the reason for a lack of an association in our study is not clear, but may again reflect the low-threshold marker of harmful alcohol use: it is possible that the adverse effect of absence of a partner for incident and persistent harmful alcohol use emerges only at heavier levels of alcohol use, or with alcohol dependence.

The recurring nature of harmful alcohol use should be considered when interpreting the results. On one hand, it could explain why the same risk factors are associated with both the incidence and persistence of harmful alcohol use: the same risk factors that potentially expose individuals to the incidence for harmful alcohol use in the first place may continue to trigger use or contribute to recurring relapses.

Evidence suggests that long-term remission from alcohol dependence often requires full abstinence, and alcohol related cues easily trigger relapse [2, 8]. For instance, cigarette smoking has shown to predict relapse after remission, and might act as a trigger to both initiation and persistence of addictive behaviours [2]. In our study, smoking was associated with both the incidence and persistence of harmful alcohol use, which supports earlier findings [13, 14],Lopez-Quintero, Pérez De Los Cobos, et al., 2011; [15]. With a similar dynamic, people having psychological distress may be more vulnerable to harmful alcohol use, but also lack mental resources needed to maintain recovery. The associations between younger age and persistent harmful alcohol use might be explained by a social environment with more exposure to alcohol consumption and fewer responsibilities, which may not only lead to harmful alcohol use, but also make it more difficult to stop. On the other hand, the recurring nature of harmful alcohol use needs to be considered when evaluating our findings, since we did not have available data on possible remissions during follow-up time, and it remains unclear whether individuals with potential longer periods of remission differ from those with continuing harmful alcohol use through the follow-up. Studies with more frequent assessments are needed to understand the potential similarities and differences of the risk factors of incidence and persistence of harmful alcohol use.

Some limitations need to be noted. Our data were observational and thus we cannot draw causal conclusions. Although we controlled for a robust set of variables potentially confounding the associations, we cannot rule out residual or unmeasured confounding. Self-reported measures of alcohol use are subject to social desirability bias, which may underestimate the rates of harmful alcohol use. The measures of harmful alcohol use were not uniform across studies, which may have introduced heterogeneity in the outcome assessment. Our study comprises data from longitudinal cohort studies, where selective loss to follow-up is inevitable. Although harmful alcohol use at baseline predicted attrition only in one of the study cohorts (BCS), it is possible that selective attrition biased the observed associations (see Supplementary Table 2). Persistent alcohol use was assessed by meeting at least one of the four CAGE criteria at baseline and at follow-up, and thus we cannot rule out possible remissions and relapses during the follow-up which was 8 years on average. It is possible that the risk factors differ between individuals with longer periods of remission during the follow-up and those who continued the harmful alcohol use throughout the follow-up, and more studies with repeated measurements are needed to evaluate such possibility in more detail. Ethnicity was not included as a sociodemographic risk factor in our analyses, because only one of the included cohorts was ethnically heterogeneous. However, given that ethnic minorities experience several psychosocial risk factors more frequently and may often have limited access to healthcare, [18], future studies should aim to include ethnicity as a potential risk factor for persistent alcohol use.

The strengths of our study include combining data of national cohorts from the United States, the United Kingdom, and Japan, and using longitudinal data in assessment of the persistence of harmful alcohol use. In addition, using subclinical measures of alcohol use enabled us to include subjects with risky yet easily unregistered or unnoticed alcohol use. Large scale evidence suggests that mortality risk associated with alcohol use increases at lower doses of weekly alcohol consumption than most of the officially defined risk thresholds are [25]. Despite the higher mortality risk, harmful alcohol users may maintain a good quality of life and continue the use regardless of the risks [24].

## Conclusions

In sum, we found that the incidence and persistence of harmful alcohol use share the same typical socio-demographic risk factors: male sex, younger age, higher educational attainment, current smoking and psychological distress. These findings suggest the need to consider targeted prevention, for example, more efficient screening of alcohol use behaviours and accessible, low-threshold prevention campaigns or programs to people with sociodemographic risk factors.

Further studies should aim to clarify these associations by adding potential biological components and ethnicity to the models in population-based studies. The role of possible relapses and remissions during longer-term persistent harmful alcohol use should also be explored in future studies.

## Supplementary Information

Below is the link to the electronic supplementary material.

Supplementary file1 (DOCX 482 KB)

## Data Availability

The data that support the findings of this study are openly available in UK Data Service at https://ukdataservice.ac.uk/ (NCDS and BCS) and Inter-university Consortium for Political and Social Research at https://www.icpsr.umich.edu/web/pages/ICPSR/index.html (WLSS, WLSG, MIDUS and MIDJA).
