# Supplementary material for: Sociodemographic risk factors for the persistence of harmful alcohol use: a pooled analysis of prospective cohort studies
Source: Soc Psychiatry Psychiatr Epidemiol. 2024 Apr 26;60(1):215–21. doi: 10.1007/s00127-024-02654-w (PMC11790781; doi:10.1007/s00127-024-02654-w)
Supplement: Supplementary file 1 — Supplementary file1 (DOCX 482 KB) [file 127_2024_2654_MOESM1_ESM.docx]

SUPPLEMENTARY MATERIAL

**Alcohol use questionnaire items**

Harmful alcohol use was evaluated at each study baseline and at follow-up with self-report questionnaires. Harmful alcohol use was assessed with the CAGE questionnaire (BCS, NCDS), or a 5-item composite (MIDUS, WLSG, WLSS), or 2-item composite (MIDJA) similar to CAGE. In the BCS and NCDS samples, the questions (CAGE) were 1) Have you ever felt you ought to *cut down* on drinking? 2) Have people *annoyed* you by criticizing your drinking? 3) Have you ever felt bad or *guilty* about your drinking? 4) Have you ever had a drink first thing in the morning (*eye*-*opener*) to steady your hands? In the MIDUS sample the questions were 1) where you ever, during the past 12 months, under the effects of alcohol or feeling its after-effects in a situation which increased you chances of getting hurt – such as when driving a car or a boat, or using knives or guns or machinery? 2) Did you ever, during the past 12 months, have any emotional or psychological problems of using alcohol – such as feeling depressed, being suspicious of people or having strange ideas? 3) Did you ever, during the past 12 months, have such a strong desire or urge to use alcohol that you could not resist it or could not think of anything else? 4) Did you have a period of a month or more during the past 12 months when you spent a great deal of time using alcohol or getting over its after-effects? 5) Did you ever, during the past 12 months, find that you had to use more alcohol than usual to get the same effect or that the same amount had less effect on you than before? In the MIDJA sample the questions were 1) during the past 12 months, how many times did you drink more than intended and 2) during the past 12 months, how many times alcohol effected work? In the WLS samples the questions included: 1) Have you ever felt bad or guilty about drinking? 2) Have people ever annoyed you by criticizing your drinking? 3) Has your drinking ever caused a problem at work? 4) Has your drinking ever created problems between yourself and spouse, children, parents, or other near relatives? 5) Have you ever gone to anyone for help about drinking?

**Supplementary Figure 1.** Associations of sex with harmful alcohol use at follow-up across the six included cohorts stratified by harmful alcohol use status at baseline

Abbreviations: OR, odds ratio; CI, confidence interval; BCS, British Birth Cohort Study; NCDS, National Child Development Study; WLSG, Wisconsin Longitudinal Study Graduate sample; WLSS, Wisconsin Longitudinal Study Sibling sample; MIDUS, Midlife in the United States Study; MIDJA, Midlife in Japan Study.

Estimates for the incidence of harmful alcohol use are from analyses among participants with no harmful alcohol use at baseline. Estimates for the persistence of harmful alcohol use are from analyses among participants with harmful alcohol use at baseline. All analyses adjusted for the other sociodemographic factors of interest and the length of follow-up (analyses in MIDUS additionally adjusted for ethnic minority status).

**Supplementary Figure 2.** Associations of age with harmful alcohol use at follow-up across the six included cohorts stratified by harmful alcohol use status at baseline

Abbreviations: OR, odds ratio; CI, confidence interval; BCS, British Birth Cohort Study; WLSG, Wisconsin Longitudinal Study Graduate sample; WLSS, Wisconsin Longitudinal Study Sibling sample; MIDUS, Midlife in the United States Study; MIDJA, Midlife in Japan Study.

Age does not vary in the NCDS (National Child Development Study) cohort so NCDS was not included in this analysis.

Estimates for the incidence of harmful alcohol use are from analyses among participants with no harmful alcohol use at baseline. Estimates for the persistence of harmful alcohol use are from analyses among participants with harmful alcohol use at baseline. All analyses adjusted for the other sociodemographic factors of interest and the length of follow-up (analyses in MIDUS additionally adjusted for ethnic minority status).

**Supplementary Figure 3.** Associations of presence of a partner with harmful alcohol use at follow-up across the six included cohorts stratified by harmful alcohol use status at baseline

Abbreviations: OR, odds ratio; CI, confidence interval; BCS, British Birth Cohort Study; NCDS, National Child Development Study; WLSG, Wisconsin Longitudinal Study Graduate sample; WLSS, Wisconsin Longitudinal Study Sibling sample; MIDUS, Midlife in the United States Study; MIDJA, Midlife in Japan Study.

Estimates for the incidence of harmful alcohol use are from analyses among participants with no harmful alcohol use at baseline. Estimates for the persistence of harmful alcohol use are from analyses among participants with harmful alcohol use at baseline. All analyses adjusted for the other sociodemographic factors of interest and the length of follow-up (analyses in MIDUS additionally adjusted for ethnic minority status). Presence of a partner was coded 0 – partnered, 1 – not partnered.

**Supplementary Figure 4.** Associations of educational attainment with harmful alcohol use at follow-up across the six included cohorts stratified by harmful alcohol use status at baseline

Abbreviations: OR, odds ratio; CI, confidence interval; BCS, British Birth Cohort Study; NCDS, National Child Development Study; WLSG, Wisconsin Longitudinal Study Graduate sample; WLSS, Wisconsin Longitudinal Study Sibling sample; MIDUS, Midlife in the United States Study; MIDJA, Midlife in Japan Study.

Estimates for the incidence of harmful alcohol use are from analyses among participants with no harmful alcohol use at baseline. Estimates for the persistence of harmful alcohol use are from analyses among participants with harmful alcohol use at baseline. All analyses adjusted for the other sociodemographic factors of interest and the length of follow-up (analyses in MIDUS additionally adjusted for ethnic minority status).

**Supplementary Figure 5.** Associations of current smoking with harmful alcohol use at follow-up across the six included cohorts stratified by harmful alcohol use status at baseline

Abbreviations: OR, odds ratio; CI, confidence interval; BCS, British Birth Cohort Study; NCDS, National Child Development Study; WLSG, Wisconsin Longitudinal Study Graduate sample; WLSS, Wisconsin Longitudinal Study Sibling sample; MIDUS, Midlife in the United States Study; MIDJA, Midlife in Japan Study.

Estimates for the incidence of harmful alcohol use are from analyses among participants with no harmful alcohol use at baseline. Estimates for the persistence of harmful alcohol use are from analyses among participants with harmful alcohol use at baseline. All analyses adjusted for the other sociodemographic factors of interest and the length of follow-up (analyses in MIDUS additionally adjusted for ethnic minority status).

**Supplementary Figure 6.** Associations of psychological distress with harmful alcohol use at follow-up across the six included cohorts stratified by harmful alcohol use status at baseline

Abbreviations: OR, odds ratio; CI, confidence interval; BCS, British Birth Cohort Study; NCDS, National Child Development Study; WLSG, Wisconsin Longitudinal Study Graduate sample; WLSS, Wisconsin Longitudinal Study Sibling sample; MIDUS, Midlife in the United States Study; MIDJA, Midlife in Japan Study.

Estimates for the incidence of harmful alcohol use are from analyses among participants with no harmful alcohol use at baseline. Estimates for the persistence of harmful alcohol use are from analyses among participants with harmful alcohol use at baseline. All analyses adjusted for the other sociodemographic factors of interest and the length of follow-up (analyses in MIDUS additionally adjusted for ethnic minority status).

| **Supplementary Table 1.** Associations of harmful alcohol use at baseline with attrition at follow-up | | | |
| --- | --- | --- | --- |
|  |  |  |  |
| Cohort | OR | 95% CI | p-value |
| BCS | 0.27 | 0.13, 0.58 | <0.001 |
| NCDS | 1.07 | 0.94, 1.22 | 0.320 |
| WLSG | 0.84 | 0.50, 1.42 | 0.516 |
| WLSS | 0.89 | 0.66, 1.20 | 0.454 |
| MIDUS | 0.91 | 0.66, 1.25 | 0.563 |
| MIDJA | 0.91 | 0.65, 1.28 | 0.589 |
| Abbreviations: OR, odds ratio; CI, confidence interval; BCS, British Birth Cohort Study; NCDS, National Child Development Study; WLSG, Wisconsin Longitudinal Study Graduate sample; WLSS, Wisconsin Longitudinal Study Sibling sample; MIDUS, Midlife in the United States Study; MIDJA, Midlife in Japan Study. | | | |
| Estimates are from logistic regression analyses adjusted for age at baseline, sex and ethnicity. | | | |
